# Thyroxine Threshold Is Linked to Impaired Outcomes in Preterm Infants

**DOI:** 10.3389/fped.2020.00224

**Published:** 2020-05-05

**Authors:** Stephanie Coquelet, Helene Deforge, Jean-Michel Hascoët

**Affiliations:** ^1^Department of Neonatology, Regional Maternity, Centre Hospitalier Regional Universitaire de Nancy, Nancy, France; ^2^EA3450- DevAH, University of Lorraine, Vandœuvre-lès-Nancy, France

**Keywords:** clinical status, extremely preterm, outcomes, threshold, transient hypothyroxinaemia

## Abstract

Transient hypothyroxinaemia of prematurity (THOP) presents as decreased free thyroxine without an increase in thyroid stimulating hormone. Thyroxine availability is important in case of premature birth, and THOP could be associated with impaired adaptation to extra-uterine life but the association of thyroxine level and clinical status has not yet been clearly defined.

**Aim:** To defined a free thyroxine threshold likely associated with neonatal clinical impairment and outcomes at age three years.

**Methods:** This retrospective cohort study included infants born before or at 28 weeks' gestation at the Regional Maternity in Nancy, France. We defined a free thyroxine threshold as a function of clinical impairment by Receiver Operating Curve analysis, validated by log likelihood iteration in binary logistic regression, in infants born from October 2008 to December 2012 and meeting neonatal clinical impairment criteria. This threshold was validated in a distinct cohort of infants born from January 2014 to December 2016. Clinical impairment was defined as assisted ventilation requirement at seven days of age plus four minor clinical disorders among heart rate, blood pressure, temperature, serum sodium and potassium, APGAR score at five minutes, vasopressor treatment and patent ductus arteriosus. The first cohort was assessed at age three years for neurodevelopmental outcomes.

**Results:** We identified a ≤10 pmol/L threshold with 85.7% sensitivity and 51% specificity. From the first and second cohorts, 196 and 176 infants respectively had available data, and 85% (97/112) and 26% (20/78) with free thyroxine ≤10 pmol/L met clinical impairment criteria. For infants with values >10 pmol/L, 41% (35/84) and 3% (3/98) from the first and second cohorts met impairment criteria. Of 147 children with available data at age 3 years, 65% (58/89) with neonatal free thyroxine ≤10 pmol/L had adverse neurodevelopmental outcomes vs. 34% (20/58) with >10 pmol/L (OR 3.55; 95% confidence interval, 1.77–7.13; *p* < 0.001).

**Conclusion:** A free thyroxine level ≤10 pmol/L in infants is associated with neonatal clinical impairment and poor outcome at age three years.

## Introduction

Thyroid function is essential for systemwide homeostasis, governing basal metabolism and organ maturation (1–3). By 10–12 weeks of gestation, the foetal thyroid gland has acquired the capacity to synthesise hormones (4). Up to 35 weeks of gestation, however, hypothalamic regulation of hormonal secretion continues to mature (5). Immaturity of the hypothalamic–pituitary axis and thyroid function often accompany premature birth (6). Term-born infants show an increase in thyroid hormone levels after birth, but preterm infants typically do not (7). Transient hypothyroxinaemia of prematurity (THOP) presents as decreased free thyroxine without an increase in thyroid stimulating hormone (8). This condition arises at about seven days of life and usually spontaneously resolves within three weeks. Because premature birth is associated with additional metabolic requirements (9), thyroxine availability is important, and THOP could be associated with impaired adaptation to extra-uterine life (10, 11). Previous studies have reported reference values for thyroid hormones according to GA (12, 13), but the association of thyroxine level and clinical status has not been clearly defined (14, 15).

The objective of this study was to define and validate a threshold value of free thyroxin level associated with neonatal morbidities in extremely premature infants. The secondary objective was to examine the association between this defined threshold of neonatal free thyroxin level and the outcome at three years of age.

## Patients And Methods

We performed a retrospective cohort study among all infants born before or at 28 weeks of gestation at our level III neonatal intensive care unit. They were excluded if they were born to a mother with thyroid or adrenal pathology, had major congenital or chromosomal abnormalities or died before seven days of life. The included infants were allocated into two groups based on year of birth. The first cohort consisted of infants born between October 2008 and December 2012, whose data we used to define a free thyroxine level threshold associated with clinical morbidities. The second cohort consisted of infants born between January 2014 and December 2016, who served as the validation cohort for the defined threshold. We also used data of children from the earlier cohort for neurodevelopmental assessments at age 3 years.

### Methods

Perinatal data were collected from infant medical records and anonymously computerised. Recorded data included gestational age, sex, birth weight, antenatal corticosteroid therapy, maternal smoking, mode of delivery, APGAR score, length of stay, and mortality. Our policy is to monitor thyroid function at seven to ten days of life in infants born at 28 weeks' GA or less who present with clinical impairment. We collected data on free thyroxine level and age at the time of measurement from our institutional database. Free thyroxine was measured using an automated immunoassay analyser, Unicel DXI 600 Access (Beckman Coulter, Brea, California).

We first analysed data of the first cohort to establish a threshold value for free thyroxine as a function of clinical impairment. We defined clinical impairment as a composite variable based on relevant neonatal morbidities, related to thyroid immaturity with regards to literature data: assisted ventilation requirement at seven days of age plus four minor clinical disorders among heart rate, blood pressure, temperature, serum sodium and potassium, APGAR score at five minutes, vasopressor treatment, and patent ductus arteriosus (7, 9, 16). Then, we used the same criteria to validate the identified free thyroxine threshold in the infants of the second cohort. The stratification of the population of this cohort, below or above the threshold, will allow calculating its sensitivity and specificity and compare them to what was found in the first cohort.

For the three-year follow-up, we collected data on infants from the first cohort. A routine follow-up program is systematically proposed to parents of prematurely born infants in our region. This follow-up program involves a standardised multidisciplinary approach to identifying neuro-psychomotor disorders requiring early support. Follow-up examiners were unaware of the child's neonatal free thyroxine status. Anthropometric data were collected from the three-year follow-up visit along with standardised psychomotor evaluation findings with selected items from the Brunet–Lezine score (17). Adverse neurodevelopmental outcomes were defined as mortality after seven days of life, motor disability including spontaneous motor activity disorders, motor impairment or cerebral palsy (18) or a poor neurodevelopmental score within the first quartile of the population.

### Statistical Analyses

Descriptive data are presented as median values and interquartile ranges. Comparison between groups was performed using Fisher's exact test for categorical variables and the Mann-Whitney *U* test for continuous variables.

The threshold value of free thyroxine associated with clinical morbidities was determined by receiver operating characteristic curve analysis defining the best compromise between sensitivity and specificity of FT4 as a function of clinical impairment (Table in Supplemental Material). That analysis was validated by log likelihood iteration in binary logistic regression. As noted, we determined this threshold in the first population and validated it in the second population with the same criteria. A cross-validation was performed using the Mann-Whitney *U* test on data within and between the two populations with bootstrap resampling.

All variables that were significantly associated with the free thyroxine threshold on univariate analysis were included in a multivariate logistic regression model with free thyroxine threshold as the dependent variable. A *p* value below 0.05 was chosen to indicate statistical significance. Statistical analyses were performed using SYSTAT 13 software (2009; Systat Software Inc., San Jose, California, USA).

### Ethical Approval

At the time of birth, parents were informed of the possible use of their children's medical information from their medical record for research purposes. All parents provided written informed consent. This study was registered at the Commission Nationale Informatique et Libertés as study number R2015-31. It received institutional review board approval on 14 February 2014 (IRB number: MRU-1403).

## Results

**Figure 1:**
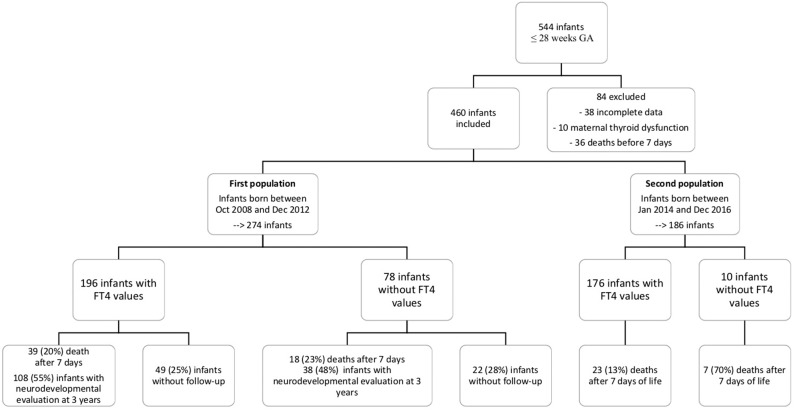
Flow chart of the population.

Data for 460 infants were included. The median GA was 27 weeks (24–28 weeks), and the median birth weight was 900 g (490–1610 g). Figure 1 shows the flowchart of the study population. In the first cohort, 274 infants met the inclusion criteria, and 186 did so from the second cohort. Demographic characteristics were similar between the groups (Table 1).

**Table 1:** Demographic and perinatal characteristics of infants born in the two time periods.

		**First period October 2008–December 2012** ***n*** **=** **274**						**Second period January 2014–December 2016** ***n*** **=** **186**		
	**Total *n* = 274**	**FT4 value** ***n*** **=** **196**			**No FT4 value** ***n*** **=** **78**			**Total *n* = 186**	**FT4 value *n* = 176**	**No FT4 value *n* = 10**
		**3 years follow-up *n* = 147**	**No Follow-up *n* = 49**	***P***	**3 years follow-up *n* = 56**	**No follow-up *n* = 22**	***P***			
**Gestational age**										
24 - 26 weeks	91 (33%)	66 (45%)	10 (20%)	0.002	14 (25%)	1 (5%)	0.05	67 (36%)	65 (37%)	2 (20%)
27–28 weeks	183 (67%)	81 (55%)	39 (80%)		42 (75%)	21 (95%)		119 (64%)	111 (63%)	8 (80%)
Birth weight, g	929 (±205)	939 (±199)	964 (±197)	0.01	952 (±199)	972 (±196)	0.05	945 (±208)	945 (±255)	947 (±10)
**Sex**										
Male	159 (58%)	87 (59%)	29 (59%)	1	32 (57%)	11 (50%)	0.6	92 (49%)	90 (51%)	2 (20%)
Female	115 (42%)	60 (41%)	20 (41%)		24 (43%)	11 (50%)		94 (51%)	86 (49%)	8 (80%)
**Antenatal steroid administration**										
Complete	125 (46%)	69 (47%)	19 (39%)	0.2	26 (46%)	11 (50%)	0.9	123 (66%)	116 (66%)	7 (70%)
Partial	102 (37%)	18 (12%)	11 (22%)		9 (16%)	4 (18%)		47 (25%)	46 (26%)	1 (10%)
No treatment	47 (17%)	60 (41%)	19 (39%)		21 (38%)	7 (32%)		16 (9%)	14 (8%)	2 (20%)
**Delivery**										
Vaginal	116 (42%)	66 (45%)	20 (41%)	0.7	22 (39%)	8 (36%)	1	95 (51%)	88 (50%)	7 (70%)
Cesarean	158 (58%)	81 (55%)	29 (59%)		34 (61%)	14 (64%)		91 (49%)	88 (50%)	3 (30%)
Maternal smoking	77 (28%)	41 (28%)	12 (24%)	0.7	14 (25%)	10 (45%)	0.1	61 (33%)	58 (33%)	3 (30%)
APGAR <5 at 5 min	66 (24%)	38 (26%)	15 (31%)	0.6	10 (18%)	3 (14%)	0.7	41 (22%)	39 (22%)	2 (20%)
Length of stay, days	63.1	68.6	72		66.2	71		67.5	67.5	65.4
Mortality	57 (21%)	39 (26%)			18 (23%)			30 (16%)	23 (13%)	7 (70%)

*FT4, free thyroxine*.

A comparison between infants whose data were analysed and those lost to follow-up showed a significant difference for GA (45 vs. 20% for infants with GA below 27 weeks, *p* < 0.001) and birthweight (842 vs. 990 g, *p* < 0.001, respectively) (Table 1).

Among the infants from the first cohort, free thyroxine measurements were available for 196 infants with a median age of 9.8 (6.1–11.0) days of life. The median free thyroxine value in this group was 9.2 (7.1–12.5) pmol/L. We found a significant correlation between low GA and low free thyroxine value, with median free thyroxine levels of 5.5 (5.1–6.2) pmol/L at 24 weeks' GA and 12.0 (8.6–15.3) pmol/L at 28 weeks' GA (*p* < 0.001). The median thyroid stimulating hormone level was 5.0 (2.1–10.2) mIU/L and did not correlate significantly with GA. Free thyroxine also was not significantly influenced by other perinatal factors, including sex, birth weight, antenatal corticosteroid therapy, maternal smoking, mode of delivery, or APGAR score.

Of the infants from the first cohort, 35 exhibited clinical impairment and had a median free thyroxine level of 6.9 (5.4–8.8) pmol/L. Clinical impairment was significantly associated with a GA <26 weeks (*p* < 0.001) and vaginal delivery (*p* = 0.008). Clinical impairment was also associated with a higher mortality rate after adjustment for GA, sex and birth weight (OR 3.39; 95% confidence interval (CI), 1.41–8.13; *p* = 0.001).

**Figure 2:**
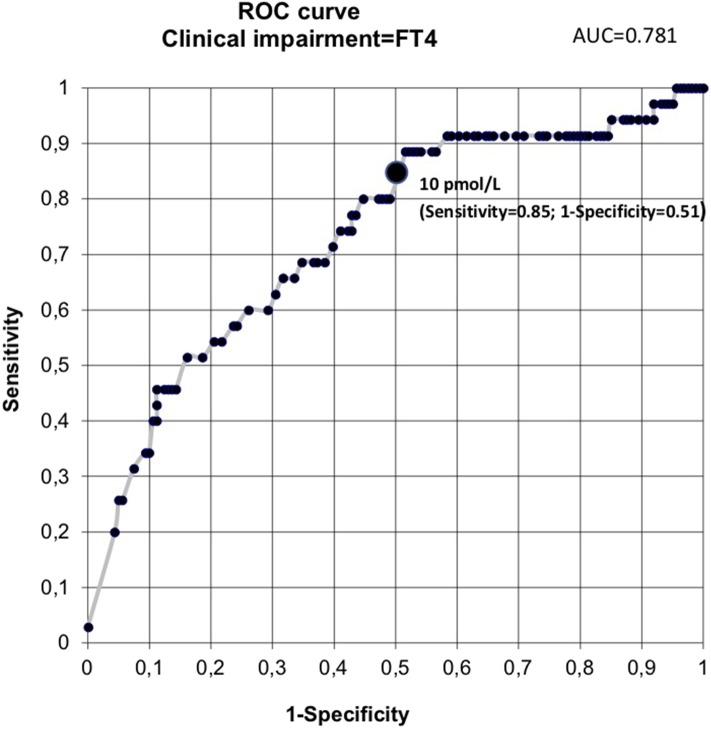
Receiver operating characteristic curve analysis. AUC, area under the curve; FT4, free thyroxine.

Receiver operating characteristic curve analysis of data from the first cohort revealed a free thyroxine value of 10 pmol/L or less as predictive of clinical impairment. This threshold had a sensitivity of 85.7%, specificity of 51%, positive predictive value of 26.8%, and negative predictive value of 94% (Figure 2). From this first cohort, 112 infants (57%) had a free thyroxine value of ≤10 pmol/L. Table 2 presents the perinatal factors significantly associated with low free thyroxine level.

**Table 2:** Perinatal factors associated with the threshold value of FT4 10 pmol/L in preterm infants born in the two time periods.

	**First period** ***n*** **=** **196**			**Second period** ***n*** **=** **176**		
	**FT4 ≤10 pmol/L *n* = 112**	**FT4 >10pmol/L *n* = 84**	***P***	**FT4 ≤10pmol/L *n* = 78**	**FT4 >10pmol/L *n* = 98**	***P***
GA 24–26 weeks	60 (53%)	16 (19%)	<0.001	43 (56%)	21 (21%)	<0.001
Birth weight (g)	815 ± 282	919 ± 287	<0.001	867 ± 273	980 ± 305	0.001
Antenatal steroid administration	92 (80%)	70 (83.3%)	0.932	68 (89%)	90 (92%)	0.311
Male sex	71 (63%)	45 (53%)	0.166	40 (52%)	48 (49%)	0.761
Vaginal delivery	59 (53%)	27 (32%)	0.004	46 (60%)	41 (42%)	0.023
Maternal smoking	23 (20%)	30 (36%)	0.020	29 (38%)	28 (28%)	0.225

*FT4, free thyroxine; GA, gestational age*.

*values are median ± interquartile range*.

Examination of the individual components of clinical impairment revealed that 97/112 infants (86%) with free thyroxine levels ≤10 pmol/L met the major criterion compared to 35/84 infants (41%) with levels >10 pmol/L. The free thyroxine threshold value was significantly associated with mechanical ventilation for more than seven days (*p* < 0.001), patent ductus arteriosus and vasopressor treatment (*p* < 0.001).

We next validated this threshold value in the infants from the second age cohort. Free thyroxine measurements were available for 176/186 infants, with a median value of 10.8 ± 6.4 pmol/L. Clinical impairment was present in 23 infants: 20/78 infants (26%) with free thyroxine levels ≤10 pmol/L compared to 3/98 infants (3%) with free thyroxine levels >10 pmol/L (*p* < 0.001). This threshold had a sensitivity of 87% and a specificity of 62% validating its value in the second cohort. The cross-validation confirmed no significant difference between the two cohorts for the clinical status of the infants (*p* = 0.7) or their free thyroxine levels (*p* = 0.2). This analysis also confirmed that the threshold of 10 pmol/L was associated with clinical impairment (*p* = 0.005) and did not differ between the two groups (*p* = 0.09). Logistic regression analysis of factors associated with FT4 ≤ 10 pmol/L showed that GA and the risk of death only were independently associated with free thyroxine threshold (OR 0.61; 95% CI, 0.38–0.95 and OR 4.94; 95% CI, 1.24–19.68 respectively), but not the other parameters included in the model such as sex, birth weight, maternal smoking or the mode of delivery (Table 3).

**Table 3:** Logistic regression analysis of factors associated with FT4 ≤ 10 pmol/L in multivariate analysis (overall model fit, *p* < 0.001).

**Parameter**	**Odds ratio**	**Standard error**	**95% confidence interval**	***P***
Death	4.938	3.484	1.239–19.685	0.024
Gestational age	0.606	0.140	0.385–0.954	0.031
Maternal smoking	0.533	0.212	0.245–1.164	0.114
Vaginal delivery	1.180	0.496	0.518–2.691	0.693
Birth weight	0.999	0.001	0.997–1.001	0.454

*FT4, free thyroxine*.

We then evaluated the long-term outcome at age three years among children in the first cohort. The mortality rate was 21% (57/274), and 68% (147/217) of living children were evaluated at age three years. Mortality rate was more important in the group with neonatal free thyroxine ≤10 pmol/L (31 vs. 5%), and more infants had no disability at three years in the group with free thyroxine >10 pmol/L (38 vs. 28%).

The median global score for the neurodevelopmental evaluation was 88 of a maximum 100, and the first quartile score was 79/100. We found that 65% (58/89) of the infants with free thyroxine levels ≤10 pmol/L had a poor outcome at age three years compared to 34% (20/58) with free thyroxine levels >10 pmol/L (OR 3.55; 95% CI, 1.77–7.13; *p* < 0.001). Table 4 presents the details of the poor outcomes.

**Table 4:** Poor outcome at 3 years of age for extremely preterm infants born between 2008 and 2012.

	**FT4 ≤10 pmol/L *n* = 112**	**FT4 >10 pmol/L *n* = 84**	***P***
Mortality^a^	35 (31%)	4 (5%)	<0.001
Motor disability^b^	2 (1.8%)	1 (1.2%)	0.7
Neurodevelopmental score at 3 years < Q1^c^	22 (20%)	16 (19%)	0.9
No adverse neurodevelopmental outcome	30 (28%)	37 (38%)	0.01
No evaluation at 3 years	23 (20%)	26 (26%)	0.09

a *Mortality: mortality >7 days of life*.

b *Motor disability: spontaneous motor activity disorders, motor impairment, or cerebral palsy*.

c *Q1: first quartile of the population, global score <79/100*.

## Discussion

THOP is considered a physiological phenomenon that resolves spontaneously (19). However, defining the threshold at which levels signal pathology and poor outcome is crucial. In this study, we found that a free thyroxine threshold of 10 pmol/L was associated with significant neonatal morbidities and a risk of poor outcomes at age three years. In a population of 107 infants born at less than 30 weeks GA, Greave et al. (13) identified a relationship between GA and free thyroxine level, with median free thyroxine levels of 6.5 pmol/L among infants born at a GA of 24–26 weeks and 11.6 pmol/L in infants born at a GA of 27–29 weeks. These data are consistent with our findings.

The origin of THOP traces to multiple factors, and a pathological threshold cannot be defined using only biological thyroid hormone levels. Iodine deficiency, hypothalamic–pituitary–thyroid immaturity, non-thyroidal illness, and drug exposure (4) may lead to low free thyroxine. Defining a pathological threshold requires accounting for the infant's clinical status and morbidities at the time of the measurement. In a population of infants born at a GA 23 to 34 weeks, Simpson et al. (20) evaluated THOP in 441 preterm infants, including the severity of illness. They showed that illness severity was indeed associated with lower serum thyroxine or triiodothyronine values at seven days of life. In our study, the definition of clinical impairment accounted for clinical events at the time of the biological measurement, which reflected the infant's state of instability. Our results are consistent with those of Simpson.

More specifically, our definition of clinical impairment focused on the organs that thyroid dysfunction most affects. Animal studies have revealed that thyroid hormones are involved in surfactant synthesis (21) and in alveolar liquid reabsorption (22). Reuss et al. (14) found in 365 preterm infants born at GA <32 weeks that severe hypothyroxinaemia was associated with an increased requirement for mechanical ventilation. In our study, the major criterion for clinical impairment was the need for mechanical ventilation at seven days of life. It was met by 86% of infants with a free thyroxine value ≤10 pmol/L vs. 41% of infants with a free thyroxine level >10 pmol/L. Thyroid hormones also influence the hemodynamic balance. Filipi et al. (23) showed that the need for dopamine therapy was correlated with lower thyroid hormone levels in a prospective observational study of 172 preterm infants. In this study, newborns never treated with dopamine had significantly higher TSH values (1.67 vs. 0.89 microU/ml *p* < 0.01) and presented a significant lower incidence of THOP (11.6% vs. 89.3% *p* < 0.01) than comparable infants receiving dopamine administration. This is consistent with the known negative effect of dopamine administration on the anterior pituitary function leading to decreased TSH excretion (23). This factor was included in the analysis. Thus, a bias related to dopamine administration is unlikely. Additionally, in 70 preterm infants born at <30 weeks' GA, Okada et al. (24) showed that the incidence of late-onset refractory hypotension was correlated with younger GA and levothyroxine supplementation.

In animal models, thyroid hormones regulate brain differentiation (25). THOP occurs in infants who do not have a fully developed brain. In 400 infants born at <33 weeks' GA, Reuss et al. (26) showed that severe hypothyroxinaemia was associated with an increased risk for cerebral palsy. We evaluated long-term outcome using a composite factor. We found a poor outcome in 65% of infants with a thyroxine value ≤10 pmol/L, compared to 35% of infants with a free thyroxine value >10 pmol/L. Mortality had a strong impact on these results, recorded for 31% of infants with free thyroxine ≤10 pmol/L vs. 5% of those with free thyroxine >10 pmol/L (*p* < 0.001). More infants survived with no disability at three years when free thyroxine was >10 pmol/L (*p* < 0.01).

The observations regarding neurological outcome have led to randomised trials of thyroxine supplementation. In a cohort of 200 preterm infants born at a GA <30 weeks, Van Wassenaer et al. (3, 27) found no significant difference between supplemented and control groups. However, within a subgroup of infants born at 25–26 weeks' GA, the treated group showed a higher mental development score at age two years compared to placebo-treated controls. Conversely, among infants born at GA >27 weeks, supplementation was associated with reduced mean mental development scores relative to controls. Of note, free thyroxine levels were not taken into account in that study. The current results warrant a prospective study evaluating the benefits of thyroxine supplementation in preterm infants with neonatal clinical impairment and free thyroxine levels ≤ 10 pmol/L.

Our study has limitations. The retrospective design carries a risk of bias because of missing data. The only difference between studied infants and those lost to follow-up was a lower birth weight in the infants whose data were included. Thus, an effect from loss to follow-up is unlikely. Despite our policy of advising monitoring from days seven to ten of life in infants with clinical impairment, the postnatal age range at monitoring was larger than expected. An effect of a few days after one week of age is unlikely. The number of infants lost to follow-up by age three years may partly explain the absence of differences among the survivors because of a lack of power. The infants included in the analysis are those who survived after seven days of life, which might be considered to confer survival bias. Before that age, early death can trace to many factors other than thyroid function. Finally, we did not incorporate the parameters associated with short-term neurologic evolution, such as intraventricular haemorrhage, which could be considered as confounding factors. We speculate that the three-year assessment is an indirect reflection of these factors.

## Conclusion

We found that a free thyroxine level of 10 pmol/L is a pathological threshold associated with neonatal clinical impairment and poor long-term outcome. Future investigations should include a prospective study evaluating the effects of thyroxine supplementation in this defined population of extremely preterm infants born at GA ≤28 weeks with clinical impairment at seven days of life and free thyroxine ≤10 pmol/L.

## Data Availability Statement

All datasets generated for this study are included in the article/Supplementary Material.

## Ethics Statement

The studies involving human participants were reviewed and approved by Commission Nationale Informatique et Libertés as study number R2015-31. Institutional review board approval on 14 February 2014 (IRB number: MRU-1403). Written informed consent from the participants' legal guardian/next of kin was not required to participate in this study in accordance with the national legislation and the institutional requirements.

## Author Contributions

SC and JMH contributed to the conceptualisation, methodology and analysis. HD performed neuropsychological infants' evaluation at 3 years of age. SC did the original draft preparation. JMH was responsible of writing, review and supervision. SC, HD, and JMH have revised and approved the submitted version of the manuscript and are responsible for the reported research and made significant contribution to the study.

## Conflict of Interest

The authors declare that the research was conducted in the absence of any commercial or financial relationships that could be construed as a potential conflict of interest.

## Abbreviations

GA: gestational age
THOP: transient hypothyroxinaemia of prematurity.
