# Supplementary material for: Thyroxine Threshold Is Linked to Impaired Outcomes in Preterm Infants
Source: Front Pediatr. 2020 May 5;8:224. doi: 10.3389/fped.2020.00224 (PMC7216369; doi:10.3389/fped.2020.00224)
Supplement: Supplementary file 1 [file Table_1.pdf]

Supplemental Table: Sensitivity and specificity of FT4 levels as a function of Clinical Impairment

SENSIT	_1MSPEC_	Se	Spe	J (Youden)	FT4 level
0.8	0.47826	80.00%	52.17%	0.32	9.5
0.8	0.47205	80.00%	52.80%	0.33	9.6
0.8	0.4472	80.00%	55.28%	0.35	9.8
**0.85714**	**0.4893**	**85.71%**	**51.07%**	**0.36**	**10**
0.88571	0.56522	88.57%	43.48%	0.32	10.1
0.88571	0.55901	88.57%	44.10%	0.33	10.2
0.88571	0.54037	88.57%	45.96%	0.35	10.3
0.88571	0.53416	88.57%	46.58%	0.35	10.4
0.88571	0.52795	88.57%	47.21%	0.36	10.5
